# Cytocompatibility of a Poly(lactic-*co*-glycolic acid) and Beta-Tricalcium Phosphate Scaffold for Applications in Bone Regenerative Medicine

**DOI:** 10.1021/acsomega.5c11611

**Published:** 2026-05-28

**Authors:** Livia S. Steinberg, Eliane Trovatti, Hernane S. Barud, Paula A Fernandes, Rafaela B Silvestre, Fernando P. S. Guastaldi, Alessandro R. Rodrigues, Agnieszka Tercjak, Marcos A. Sabino, Clóvis A. Ribeiro, André C. Amaral

**Affiliations:** † Graduate Program in Biotechnology, 74374 Universidade de Araraquara (UNIARA), Rua Carlos Gomes, 1217, Araraquara, São Paulo 14801-040, Brazil; ‡ Division of Oral and Maxillofacial Surgery, Department of Surgery, Massachusetts General Hospital, Harvard School of Dental Medicine, 50 Blossom St, Boston, Massachusetts 02114, United States of America; § Department of Mechanical Engineering, São Carlos School of Engineering - Universidade de São Paulo (USP), Avenida Trabalhador São-Carlense, 400, São Carlos, São Paulo 13566-590, Brazil; ∥ Department of Chemical and Environmental Engineering, Group `Materials+Technologieś (GMT) - Universidad del País Vasco/Euskal Herriko Unibertsitatea (UPV/EHU), Plaza Europa 1, Donostia -San Sebastián 20018, Spain; ⊥ B5IDA research group, Department of Chemistry, Universidad Simon Bolivar, Caracas AP-89000, Venezuela; # Department of Analytical Chemistry, Physical-Chemistry, and Inorganic, Institute of Chemistry of Araraquara, Universidade Estadual Paulista (UNESP), Rua Professor Francisco Degni, 55, Araraquara, São Paulo 14800-900, Brazil

## Abstract

Critical-size bone defects (CSDs) represent a clinical challenge as they exceed the bone’s intrinsic regenerative capacity. Given the limitations of conventional therapies, regenerative medicine offers strategies based on scaffolds to promote tissue reconstruction and restore bone function. This study aimed to perform the physicochemical, morphological, topographical, and surface mechanical characterization of a poly­(lactic-*co*-glycolic acid)/beta-tricalcium phosphate (PLGA/β-TCP) scaffold and to assess its in vitro cytocompatibility profile. To this end, PLGA/β-TCP scaffolds were designed and fabricated using the same raw materials and manufacturing process as a commercially available product. Characterization was performed by X-ray diffraction (XRD), Fourier-transform infrared spectroscopy (FT-IR), thermogravimetric (TG), and differential scanning calorimetry (DSC) analyses, optical profilometry (OP), scanning electron microscopy (SEM), atomic force microscopy (AFM), and PeakForce quantitative nanomechanical mapping (QNM). Cytocompatibility was assessed using the MG-63 osteogenic cell line. The incorporation of β-TCP significantly increased the surface roughness and stiffness of the scaffold, as evidenced by elevated values across all analytical parameters. The resulting architecture was more complex, with a predominance of rounded peaks and valleys and a larger surface area. These topographical features are directly associated with the enhanced cytocompatibility observed for the same sample, particularly within the first 24 h of analysis, indicating improved cell adhesion of the biomaterial. It is worth noting that the topography of the 3D-printed PLGA/β-TCP structures was analyzed in detail using unconventional parameters, providing a more comprehensive understanding of the surface behavior and its biological implications. This detailed approach revealed the positive influence of these characteristics on cytocompatibility, an essential aspect for osseointegration. These findings contribute to the understanding of the biological performance of this composite and provide support for its application in tissue engineering and bone regenerative medicine.

## Graphical abstract

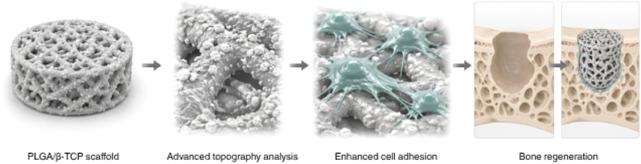

## 1 Introduction

Bone tissue is characterized by its intrinsic ability to remodel and renew throughout an individual’s life continuously, as well as to adapt to mechanical and physiological stimuli. Through this same mechanism, bone cells are capable of repairing injuries without the need for external intervention.^1^ However, in certain situations, the extent of the injury may exceed the tissue’s regenerative capacity, resulting in structural and functional impairment of the musculoskeletal system and, consequently, a decline in the individual’s quality of life.^2^ These injuries are referred to as critical-sized bone defects (CSDs) and are defined by substantial bone loss, commonly arising from high-impact fractures, extensive infections requiring aggressive debridement, blast injuries, or tumors.^3^ In such cases, the native cellular machinery is often insufficient to restore the damaged tissue, making additional intervention through the implantation of bone substitutes necessary.^4^

Bone substitutes, also referred to as bone grafts, are required to exhibit osteoconductive properties—enabling new bone tissue to be deposited along their surface in the presence of preexisting bone as a source of osteogenic cells; osteoinductive properties—promoting the differentiation of mesenchymal stem cells into bone-forming cells; and osteogenic properties—directly supporting the formation of new bone tissue through the activity of osteoblasts. These grafts can be classified based on their origin: autografts, harvested from the patient’s own healthy bone; allografts, obtained from donors of the same species; xenografts, derived from other species; and synthetic grafts, which are made from biocompatible materials. Although autografts are considered the gold standard in the treatment of CSDs due to their inherent biological advantages, their application is limited by the restricted availability of donor bone, increased morbidity associated with a second surgical site, and the heightened risk of infection and functional impairment—factors that make this option less desirable in certain clinical scenarios.^5,6^

In this context, bone regenerative medicine (BRM) aims to address the challenge of CSDs by providing a safer and more effective alternative to conventional treatment. This approach involves the use of biocompatible and biodegradable materials that, when combined with cells, form a scaffold capable of replicating the native bone microenvironment. Scaffold-based strategies have become a central pillar in the therapeutic approach to CSDs, as they provide a three-dimensional temporary framework capable of guiding cell adhesion, proliferation, and differentiation while supporting tissue ingrowth and vascularization. In guided bone regeneration (GBR), scaffolds are designed to mimic key structural and physicochemical features of native bone, enabling mechanical support, spatial control of tissue formation, and synchronized degradation with new bone deposition. A wide diversity of biomaterials, including biodegradable polymers, bioactive ceramics, and composite systems, has been explored for scaffold fabrication, with composition, architecture, and degradation behavior playing a critical role in modulating mechanical performance, biological response, and overall regenerative outcomes.^7–10^

Poly­(lactic-*co*-glycolic acid) (PLGA) is a biodegradable polyester copolymer composed of lactic and glycolic acids, known for its semicrystalline structure and thermoplastic behavior. As a biodegradable polyester, PLGA undergoes hydrolytic degradation through the cleavage of ester bonds, releasing byproducts that are resorbed by the body, which confers its bioresorbable character and makes it a suitable material for the fabrication of temporary bone grafts. Compared with its homopolymers, polyglycolic acid (PGA) and polylactic acid (PLA), PLGA offers the advantage of a more controlled degradation rate, allowing for a gradual and safe replacement by native bone tissue. Accordingly, PLGA has been widely employed in the development of sutures, membranes, and scaffolds owing to its favorable mechanical strength, processability, and tunable degradation profile. However, despite its bioresorbable nature, PLGA degradation can lead to the release of acidic byproducts that lower the local pH, potentially triggering inflammatory responses, protein denaturation, and degradation of the organic phase of the bone matrix.^11–17^

Beta-tricalcium phosphate (β-TCP) is widely used and is a calcium phosphate ceramic closely related to the inorganic component of the bone matrix. Unlike hydroxyapatite (HA), β-TCP is resorbed through acidification mediated by osteoclasts, allowing it to be gradually replaced by newly formed bone. Through this mechanism, β-TCP is characterized as an osteoinductive material, and its osteoconductive properties have also been well documented. Due to these characteristics, β-TCP plays a significant role in reconstructive surgeries. Nevertheless, the formulation of biomaterials using pure β-TCP may present limitations, particularly related to its low mechanical strength.^18–22^

In this context, a composite material formed by the combination of PLGA and β-TCP represents a relevant example of biodegradable polyester/ceramic composites, which have emerged as an important materials class for scaffold-guided bone regeneration. These systems combine the controlled degradation and processability of aliphatic polyesters with the osteoconductive and reinforcing contributions of calcium phosphate ceramics. In this framework, PLGA provides structural support and resorption control, while β-TCP contributes bioactive cues, mechanical reinforcement, and osteoconductivity. Acting synergistically, both phases mitigate each other’s inherent limitations, resulting in composites with favorable mechanical properties, a well-controlled degradation rate, and excellent biocompatibility. Consequently, PLGA/β-TCP composites hold significant potential for medical engineering applications, including the development of scaffolds for bone grafts and fixation devices, such as screws for reconstructive surgeries.^23^

Among the various scaffold manufacturing methods used in tissue engineering, 3D printing is the most widely employed. This additive manufacturing technique involves the layer-by-layer deposition of material, resulting in a customized final product with good resolution and low production cost. Fused Deposition Modeling (FDM) is a 3D printing technique that relies on the heating of biomaterials, typically in the form of granules or filaments, which are then deposited layer-by-layer and subsequently cooled. This approach allows for the adjustment of printing parameters such as the number and height of layers, printing grid angle, and infill thickness—all of which influence subsequent biological interactions,^22,24,25^

Based on research demonstrating the potential benefits of using these materials in the composition of scaffolds for bone substitute applications, composite biomaterials combining both have been proposed and are currently approved for clinical use by regulatory agencies in countries such as the United States and Brazil, among others. Although these materials are routinely employed, particularly considering the scaffold manufacturing methods, the precise impact of physicochemical, topographical, and surface mechanical properties—each specific to a given product—on therapeutic efficacy and safety remains insufficiently defined.

In summary, although autologous grafts can be commonly used for the treatment of SCDs, they are associated with considerable limitations, often presenting a disproportionate number of disadvantages relative to their benefits. In this context, the development of PLGA and β-TCP scaffolds through a multifilament 3D printing process using Fused Deposition Modeling (FDM) technology represents a potentially innovative approach for the construction of biological supports. This is particularly due to their unique physicochemical composition, architecture, surface topography, and mechanical profile, features not completely explored. Therefore, this study aimed to perform a comprehensive physicochemical, morphological, topographical, and surface mechanics characterization of two-dimensional scaffolds composed of poly­(lactic-*co*-glycolic acid) (PLGA) and beta-tricalcium phosphate (β-TCP) and to evaluate their cytocompatibility profile, in order to understand the biological performance of this composite and provide support for their application as a strategy in BRM.

## 2 Materials and Methods

### 2.1 PLGA and PLGA/β-TCP Scaffolds

The pure PLGA and PLGA/β-TCP composite structures were designed using computer-aided design (CAD) software, resulting in a disc-shaped geometry with a diameter of 20 mm, a solid base, and a 2 mm high rim. After defining the design parameters, the structures were manufactured by fused deposition modeling (FDM) using a multifilament 3D printer (Raise3D Pro2 Series, DMC Equipament’s), employing PLGA filaments blended with β-tricalcium phosphate (β-TCP). All raw materials, processing steps, and manufacturing conditions applied in the fabrication of the experimental scaffolds are identical to those used in the production of the commercially available bone graft substitute VITAGRAFT. The PLGA/β-TCP filaments were previously produced by extrusion under controlled industrial conditions. The development of these structures was carried out in collaboration with the Nupen Institute and DMC Equipament’s. Detailed proprietary parameters related to filament formulation and extrusion processing cannot be fully disclosed due to industrial confidentiality agreements, as appropriately noted by the reviewer.

### 2.2 Physical–Chemical Characterization

#### 2.2.1 X-ray Diffraction (XRD)

Microstructural analysis of the materials was performed using XRD. For this purpose, a diffractometer (SIEMENS, D5000, Universidade de Araraquara (UNIARA), Araraquara—SP, Brazil) was employed, operating over an angular range from 0° to 50°, with a CuKα radiation source. The operating voltage and current were set at 30 kV and 20 mA, respectively.

#### 2.2.2 Fourier Transform Infrared Spectroscopy (FT-IR)

The chemical composition of the materials was evaluated using a PerkinElmer spectrometer (Spectrum 100, located at the Renato Archer Information Technology Center – CTI, Campinas—SP, Brazil) in attenuated total reflectance (ATR) mode. FTIR spectra were obtained in the range of 4000–450 cm^–1^, with a resolution of 4 cm^–1^ and 16 scans per sample.

#### 2.2.3 Thermogravimetric (TG) and Differential Scanning Calorimetry (DSC) Analyses

Thermogravimetric and Differential Scanning Calorimetry analyses were performed using a TA Instruments SDT Q600 equipment (Universidade de Araraquara (UNIARA), Araraquara—SP, Brazil). Sample masses of approximately 6 to 8 mg were placed on a sealed aluminum tray. The experiment was conducted under a nitrogen atmosphere, with a temperature range from 29 to 800 °C and a heating rate of 10 °C·min^–1^.

#### 2.2.4 Wettability

Wettability analysis was performed by measuring the contact angle of the samples using a tensiometer Theta (Attension Biolin, University of São Paulo (USP), São Carlos—SP, Brazil) at the Center for Instrumental Chemical Analysis (CAQI/QSC), University of São Paulo—USP, São Carlos. The measurement involved the deposition of a drop of distilled water (1 mL) onto the surface of neat PLGA and PLGA/β-TCP composite scaffold.

### 2.3 Morphological Characterization

The morphological characterization of the neat PLGA and PLGA/β-TCP scaffolds was performed using scanning electron microscopy (SEM) with a field emission microscope (FEG) (MIRA3 XMU, TESCAN, Renato Archer Information Technology Center—CTI, Campinas—SP, Brazil). The acceleration voltage ranged from 4 to 10 kV, with a spot size of 4 (0.2 nm), using a secondary electron (SE) detector. To avoid charging effects, the samples were coated with gold using BALZERS—SCD 050 (BALTEC) sputtering, with a current of 50 mA, a sputtering time of 30 s, argon as the operating gas, and a 0.05 mbar working pressure. This process resulted in a gold layer approximately 10 nm thick. SEM analyses were performed in collaboration with the Renato Archer Information Technology Center (CTI).

### 2.4 Topographic Characterization

This section describes all of the roughness and surface texture parameters used in the study. It is important to note that the analysis techniques applied to these parameters were optical profilometry (OP) and atomic force microscopy (AFM), which allow for evaluations at the micrometer and nanometer scales, respectively.

#### 2.4.1 Optical Profilometry (OP)

To analyze surface topography and geometry at the microscale, the printed scaffolds were examined using an optical 3D profilometer (Veeco, Wyko NT1100 model, University of São Paulo (USP), São Carlos—SP, Brazil) with a *Z*-resolution of less than 1 nm and an XY-resolution between 1 and 2.5 mm. The analysis was conducted at the Precision Engineering and Manufacturing Innovation Group (IMEP) of the São Carlos School of Engineering (EESC), University of São Paulo (USP), under the supervision of Prof. Dr. Alessandro Roger Rodrigues.

#### 2.4.2 Atomic Force Microscopy (AFM)

For nanoscale topographical analysis, an atomic force microscope (Dimension Icon Bruker equipped with a Nanoscope VI controller, Universidad del País Vasco/Euskal Herriko Unibertsitatea, Donostia-San Sebastián, Spain) was used in tapping mode, employing a silicon cantilever coated with aluminum (tip radius ∼10 nm), with measurements performed at 512 lines under room temperature conditions.

### 2.5 Characterization of the Mechanical Properties

To evaluate the surface mechanical properties of scaffold surfaces, PeakForce Quantitative Nanomechanical Mapping (QNM) of AFM (Dimension Icon Bruker equipped with a Nanoscope VI controller, Universidad del País Vasco/Euskal Herriko Unibertsitatea, Donostia-San Sebastián, Spain) was used. This technique allows the mapping of nanomechanical parameters through force–distance curves. The Derjaguin–Muller–Toporov (DMT) model was employed to calculate Young’s modulus, which is derived from the adhesive forces between the cantilever and the sample.

### 2.6 In Vitro Biological Tests

#### 2.6.1 Culture of Human Osteosarcoma Cells (MG-63)

Human osteosarcoma cells were obtained from the Rio de Janeiro Cell Bank (UFRJ—Rio de Janeiro, Brazil) and maintained under standard culture conditions in an incubator at 37 °C, with a humidified atmosphere containing 95% air and 5% CO_2_. The cells were cultured in bottles with Dulbecco’s Modified Eagle Medium (α-MEM—Vitrocell), supplemented with 10% fetal bovine serum (FBS—Vitrocell), penicillin (100 U/mL—Vitrocell), and streptomycin (100 μg/mL—Vitrocell). Upon reaching 80% confluence, the cells were subjected to subculture procedures, in which they were removed from the bottle using a trypsin/EDTA solution (0.25%—GIBCO). After centrifugation, a pellet was formed, fractionated, and then resuspended in culture medium before being reseeded in new bottles. This process, known as cell passage, was repeated three times until the cells were ready for experiments using PLGA and PLGA/β-TCP scaffolds.

#### 2.6.2 Cytocompatibility Test

For the in vitro analysis of cytocompatibility of scaffolds composed of pure PLGA and PLGA/β-TCP, focusing on cell adhesion and viability, triplicate samples were used in the resazurin–resorufin colorimetric assay. The scaffolds were placed in a 24-well plate, and 100 μL of a cell suspension at a concentration of 1 × 10^5^ cells/mL was seeded onto each scaffold. Subsequently, the total volume in each well was completed with 300 μL of DMEM supplemented with 10% fetal bovine serum (FBS), ensuring full coverage of the scaffold. The plate was then incubated at 37 °C in a humidified atmosphere containing 5% CO_2_ for 6 h to allow for initial cell adhesion. After this period, 1 mL of supplemented DMEM was added to each well, and the plate was returned to the incubator. Next, 24 h after the start of the culture, 400 μL of a 0.01% (v/v) resazurin solution was added to each well, protected from light, and the plate was incubated again for 4 h. Then, the contents of each well were transferred to a mirror plate and stored in a refrigerator until analysis. The wells were then washed with 500 μL of phosphate-buffered saline (PBS) to remove any resazurin residue, followed by the addition of 1 mL of DMEM supplemented with 10% FBS, and the plate was incubated for another 24 h. After this period, the resazurin staining step was repeated. At the end of the 48 h culture period, the mirror plate was read using a fluorescence spectrophotometer, with an excitation wavelength of 560 nm and an emission wavelength of 590 nm. The cell culture plate was then stored in a refrigerator until further analysis.

To qualitatively assess cell morphology and density, PLGA and PLGA/β-TCP structures were analyzed by fluorescence microscopy. Initially, cells were fixed according to the following protocol. The culture medium was removed from each well, which were then washed once with phosphate-buffered saline (PBS). Subsequently, 4% paraformaldehyde was added to each well, and the plate was kept protected from light at room temperature for 30 min. After fixation, the solution was removed, the wells were rinsed with distilled water and then filled with PBS. The plate was stored at 4 °C until the staining procedure.

For staining, two fluorescent reagents were used: 4′,6-diamidino-2-phenylindole (DAPI), which binds to cell nuclei, and Fluorescein (FITC), which labels the cytoskeleton. Initially, PBS was removed from each well, and DAPI was added until the samples were fully covered. The plate was then kept protected from light for 10 min. After this period, the wells were rinsed three times with distilled water, and PBS was added again while awaiting FITC staining. After 1 week, PBS was removed and FITC was added to each well until the samples were covered. As with the previous step, the plate was stored protected from light for a few minutes. Afterward, the reagent was removed, the samples were rinsed three times with distilled water and then submerged in PBS again and stored at 4 °C until analysis by fluorescence microscopy using a Leica MDI8 microscope.

### 2.7 Statistics

Statistical analysis was performed using descriptive statistics, followed by two-way analysis of variance (two-way ANOVA) and Tukey’s multiple comparison posthoc test. A significance level of 5% (*p* < 0.05) was considered.

## 3 Results and Discussion

### 3.1 Physicochemical Characterization

#### 3.1.1 X-ray Diffraction (XRD)

The XRD spectra for PLGA, β-TCP, and PLGA/β-TCP samples are shown in Figure 1. When analyzing PLGA using X-ray diffraction (XRD), the typical pattern expected is the absence of distinct peaks, indicating an amorphous structure. However, crystallization can be induced by isothermal annealing at 115 °C for at least 60 min. This treatment^26^ led to an increase in the degree of crystallinity (21%). In contrast, the PLGA and composite material used in this study had degrees of crystallinity (*CrI*) of 27.6% and 77.0%, respectively, as determined by eq 1,^27^ where *A*_*C*_ corresponds to the area of crystalline peaks and *A*_*T*_ represents the total area of the diffractogram:

1 $$CrI\;\left(\%\right)=A_{C}/A_{T}$$

**Figure 1:**
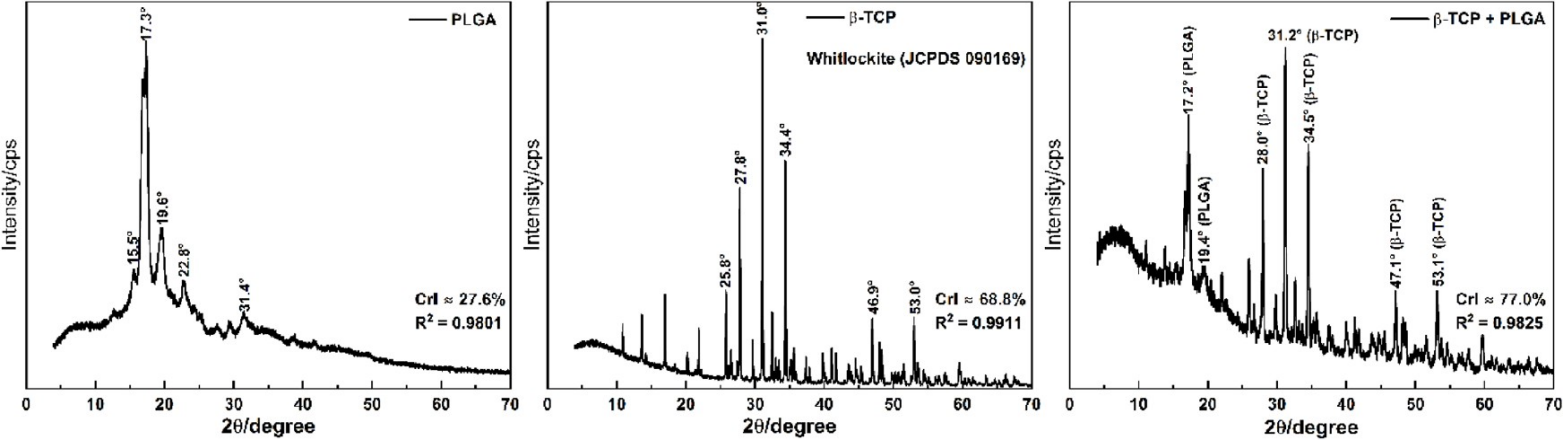
X-ray diffraction patterns of powder β-TCP, neat PLGA, and PLGA/β-TCP composite scaffolds.

As shown in Figure 1, PLGA displays characteristic XRD peaks at 15.5°, 17.3° (main), 19.6°, 22.8°, and 31.4°. Regarding β-TCP, it exhibits a Whitlockite-type structure (JCPDS 090169),^28^ with characteristic peaks at 25.8°, 27.8°, 31.0° (main), 34.4°, 46.9°, and 53.0°. As for the PLGA/β-TCP composite, the presence of both β-TCP and PLGA peaks can be observed.

#### 3.1.2 Fourier Transform Infrared Spectroscopy (FT-IR)

Figure 2 shows the general FTIR spectra obtained for each sample, with emphasis on the fingerprint region, where spectral changes are typically observed. Initially, the characteristic infrared (FTIR) bands of PLGA reflect the chemical composition of its constituent monomers, lactic and glycolic acids. The most prominent and diagnostic band of the polymer phase occurs at approximately 1750 cm^–1^, which is attributed to the carbonyl stretching (−C=O) found in the ester groups of the polymer chain. Additionally, the spectrum features a set of intense peaks in the region of 1088 cm^–1^ and 1184 cm^–1^, corresponding to the stretching of carbon–oxygen (−C–O−) bonds. Other smaller vibrations, such as the one observed at 1269 cm^–1^, are also associated with the ester group, while the peak at 1040 cm^–1^ completes the fingerprint of the material’s chemical structure.^29^ In the context of the study, these control bands serve as a reference to monitor the matrix phase of this composite in relation to the polymer’s chemical bonds.^30^

**Figure 2:**
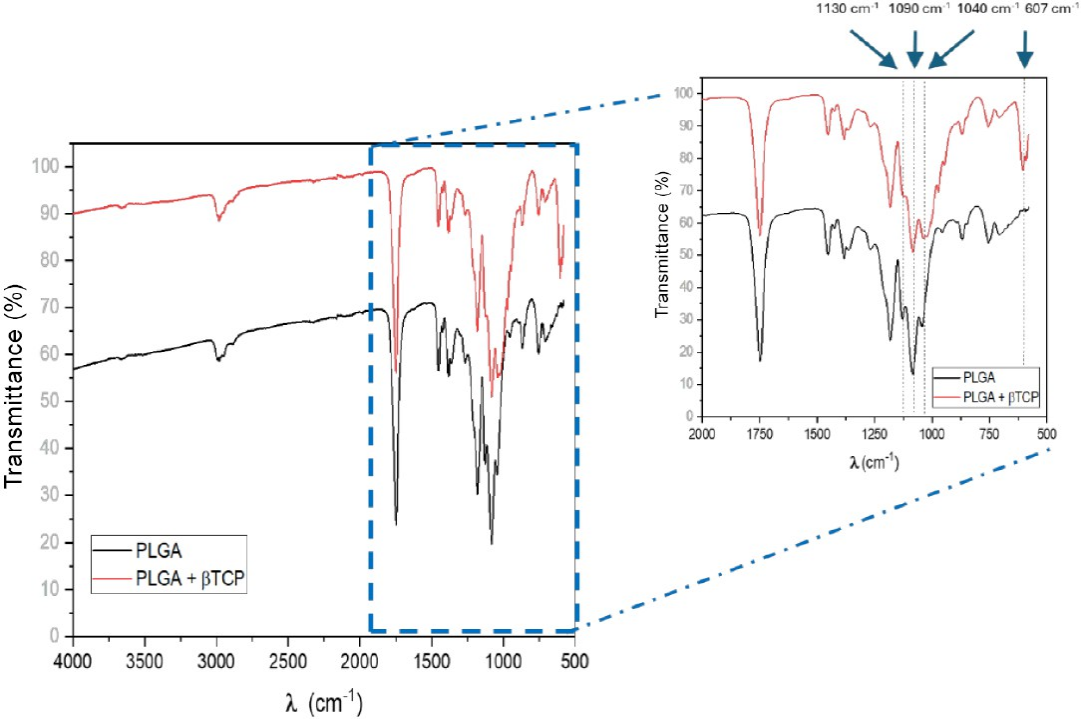
FTIR spectrum of neat PLGA (black line) and PLGA/β-TCP (red line) composite scaffolds.

Considering that the polymer/ceramic sample has a 70/30 composition, the PLGA bands appear more intense than those of β-TCP. Nevertheless, it is well known that ceramics commonly exhibit vibrational modes in the 1500–500 cm^–1^ region. Notably, the characteristic spectrum of the PO_4_^3–^ group was observed with a peak at 607 cm^–1^, indicating the presence of β-TCP.^31^

#### 3.1.3 Thermogravimetric (TG) and Differential Scanning Calorimetry (DSC) Analyses

In Figure 3(a), the thermal behavior of the samples under a nonthermo-oxidative environment is summarized for each phase, namely the PLGA copolymer, β-TCP, and the PLGA/β-TCP composite. In the case of the ceramic phase, we see that within the evaluated temperature range, there is no mass loss due to its high thermal resistance, which varies according to the literature^32^ but generally occurs at elevated temperatures above 700 °C; the slight mass loss observed in TGA is associated with the release of volatile components and the structural decomposition of this compound.

**Figure 3:**
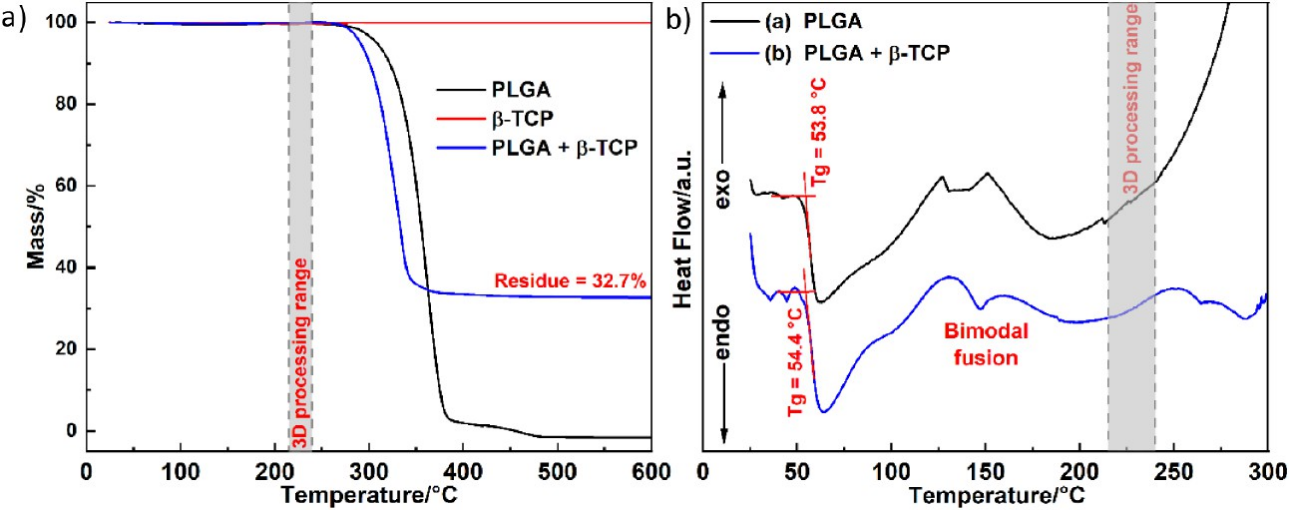
TGA (a) and DSC (b) curves for β-TCP powder, neat PLGA, and PLGA/β-TCP composite scaffolds.

For the polymeric phase, PLGA copolymer, it is observed that decomposition occurs at temperatures close to 325–327 °C and given that the polymer has a high molecular weight, the thermal decomposition process occurs due to the degradation of ester groups.^33^ Thus, as noted between 380 and 440 °C, a small hump appears, indicating this final phase of decomposition, related to a more complete degradation of the polymer and the release of volatile gases; finally, only ashes associated with carbon characteristic of organic compounds remain (*T* > 500 °C). In the literature, it is reported that the decomposition temperature of this PLGA ester-type copolymer depends on the composition ratio of the copolymers (i.e., the ratio between the glycolic acid and the lactic acid that compose its structure),^34^ and it is in the range of 250–300 °C, which coincides with what was observed in our results.

When the analyzed system corresponds to the composite material (PLGA/β-TCP), the results demonstrate that the formulation used has effectively 32–33% of the ceramic phase and 67–68% of the polymeric phase. This information is relevant because this composition may be responsible for the mechanical, morphological, and chemical properties of these scaffolds, which are being studied in this work. It is also noticeable that in this case, the thermogravimetric process is advanced, with the onset of the process occurring at approximately 300 °C. This happens because the heating in the ceramic is transferred through thermal conduction to the organic phase, facilitating its decomposition.^35^ This decomposition is rapid and is only halted by the presence of β-TCP above 360 °C. These findings are consistent with what is reported in the literature for composites with a similar formulation.^36^

When the obtained results in Figure 3(b) are analyzed, it is observed that for the PLGA copolymer, there seems to be a small but broad hump that could indicate that this copolymer is experiencing a bimodal fusion between 130 and 170 °C, which is also consistent with what is reported in the literature.^37^ Glycolic acid tends to hinder the crystallization of the chains, decreasing the overall crystallinity of the copolymer. When the proportion of lactic acid increases, PLGA tends to be more crystalline, and its melting temperature may approach that of poly­(lactic acid) (PLA), which is around 160–180 °C; not to mention that the molecular weight also influences this determination.

The addition of a certain proportion of β-TCP to the PLGA matrix leads to the formation of a composite in which the ceramic particles “trap” the polymeric chains, preventing their reorganization into crystalline structures (see Figure 3(b), black line). This results in a system with the appearance of being amorphous, which does not exhibit a melting temperature but maintains the glass transition temperature, *T*_g_ (53–54 °C), as its main thermal property,^38^ which does not seem to have a significant difference when comparing the polymer alone to the composite, as shown in Figure 3(b).

#### 3.1.4 Wettability

The contact angles of the samples are graphically represented in Figure 4. The neat PLGA exhibited a contact angle of 28–30°, whereas the PLGA/β-TCP composite showed a higher value of 76°. The pure PLGA scaffold displayed a lower contact angle, indicating a more hydrophilic surface, which favors surface protein adsorption and, consequently, cell adhesion.^25^ However, the PLGA/β-TCP composite formed a larger angle, yet it still qualifies as a hydrophilic material, as it remains below the hydrophobicity threshold value.^39^ Despite these results, the composite material sample exhibited higher cell viability values, as will be shown in the following sections, particularly during the cell adhesion phase—reinforcing the notion that hydrophilicity is not the sole determinant of cell–substrate integration.

**Figure 4:**
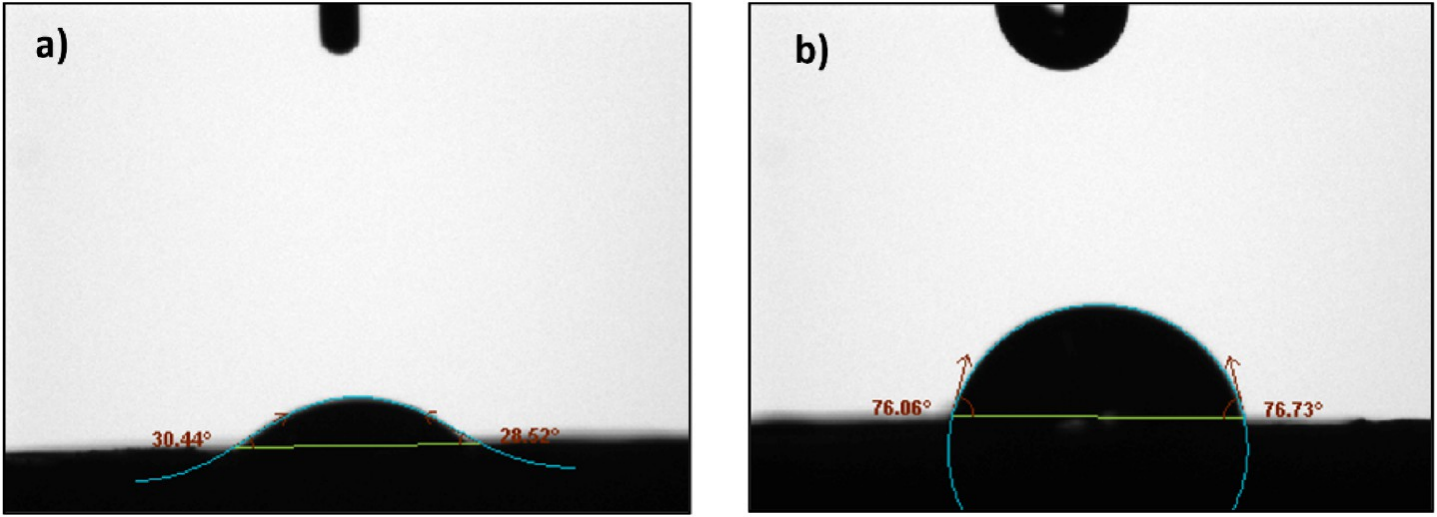
Contact angle of neat PLGA (a) and PLGA/β-TCP composite (b) scaffolds.

### 3.2 Morphological Characterization

Initially, a certain difference in surface roughness was observed between the edges and the center of both materials, resulting from the printing process, as shown in Figure 5. In this context, the central region of the PLGA/β-TCP scaffold appeared even rougher than that of PLGA, with the formation of well-defined spaces between the filaments. In contrast, as confirmed by SEM measurement, the surface of the PLGA sample appeared smoother and more uniform, without deep markings between the filaments. It is known that neat β-TCP exhibits granular behavior and contributes to surface roughness, thereby influencing the microstructural characteristics of the sample.^14^ This supports the SEM findings, in which β-TCP was shown to modify the morphology and topography of the materials. The roughness results, also assessed via optical profilometry, are discussed in greater detail in Section 3.3.

**Figure 5:**
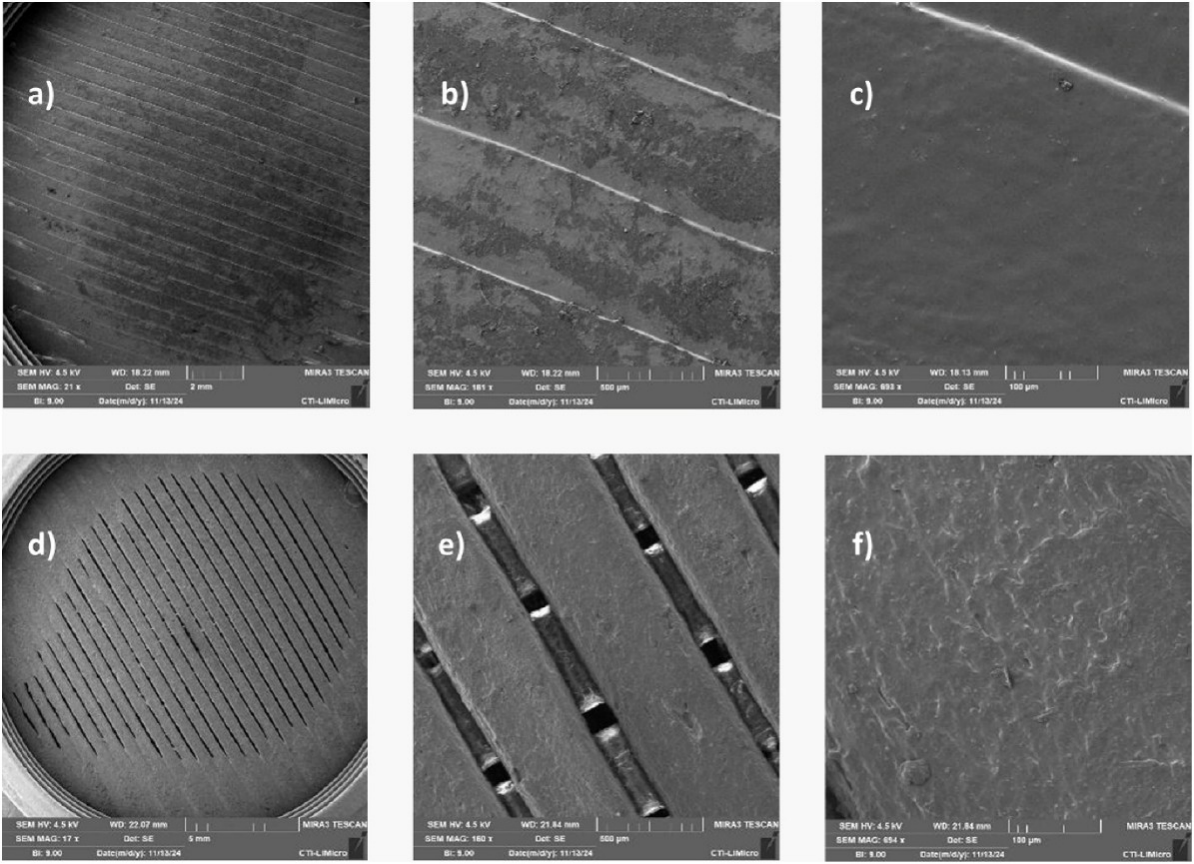
Scanning electron microscopy images revealing the topography of neat PLGA (a, with a magnification of 21×; b, with a magnification of 161×; c, with a magnification of 693×) and PLGA/β-TCP composite scaffolds (d, with a with magnification of 17×; e, with a magnification of 160×; f, with a magnification of 694×).

### 3.3 Topographic Characterization

For this analysis, a projected rectangular area of 1.08 mm^2^ (two-dimensional projection on a plane) of the fabricated topography was defined. The results revealed differences in surface roughness due to the formation of peaks and valleys on the filament surfaces.

The topographical analysis of the neat PLGA and PLGA/β-TCP scaffolds, on a micrometric scale, was performed using optical profilometry and is graphically presented in Figure 6. As expected, both neat PLGA and PLGA/β-TCP composites exhibited plateau regions corresponding to the deposited filaments, and slot regions corresponding to the gaps between them. This can be attributed to the printing trajectory—known as the raster pattern—and to the intrinsic characteristics of the materials.

**Figure 6:**
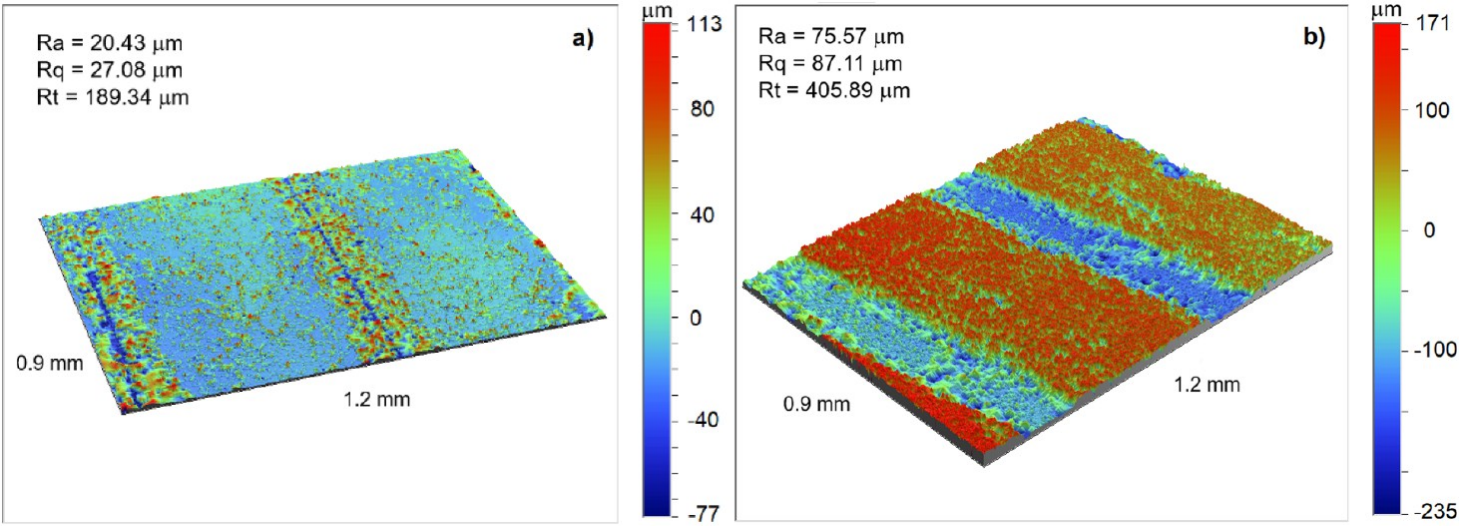
Three-dimensional surfaces of 3D-printed neat PLGA (a) and PLGA/β-TCP composite (b) scaffolds.

From a structural analysis perspective, the PLGA/β-TCP composite scaffold exhibited wider and deeper valleys (2D roughness) compared to neat PLGA (plateau areas), in addition to the slots with widths of 188 and 25 μm aforementioned and presented in Figure 7. This result may be associated with the presence of β-TCP granules dispersed throughout the scaffold, as well as with possible changes in the thermophysical properties induced by the incorporation of this ceramic into the polymer matrix.^40^

**Figure 7:**
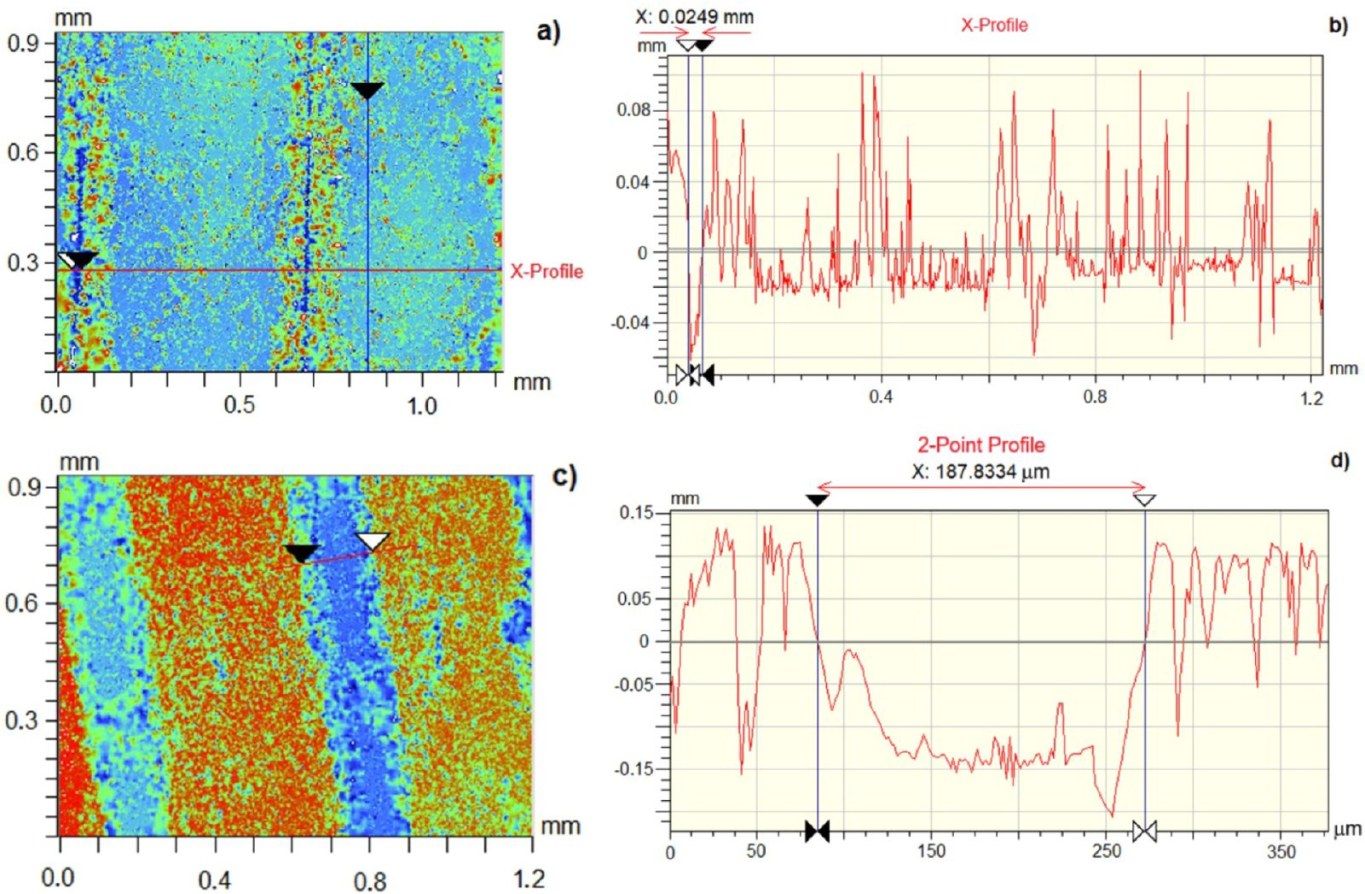
Two-dimensional surfaces and channel widths of neat PLGA (a,b) and PLGA/β-TCP composite scaffolds (c,d).

Such modifications likely enhanced the dimensional stability of the filament after deposition, contributing to a reduction in filament deformation during the cooling process. Although the initial intent was to characterize surface properties, these thermophysical changes led to the formation of channels between filaments, thereby increasing the material’s porosity. It is also important to consider the probable interconnectivity between these channels, established through layer overlap. According to current literature, critical parameters for selecting an ideal scaffold for bone grafts include porosity, pore morphology, and pore interconnectivity. Bose and collaborators emphasize that a minimum pore size of 150 μm is necessary for the development of bone substitutes,^41^ as it facilitates nutrient transport and cell infiltration during the scaffold’s integration with host tissue.

The following 3D or areal parameters reveal surface amplitude characteristics, allowing for a more in-depth characterization of the scaffolds. Considering the classic micrometric-scale roughness parameters presented in Table 1 (ISO 25178-2, 2012),^42^ the PLGA/β-TCP composite scaffold showed significantly higher values for arithmetic mean roughness (*S*_a_), root-mean-square roughness (*S*_q_), and maximum height roughness (*S*_z_), reaching increases of 270%, 222%, and 143%, respectively, compared to neat PLGA.

**Table 1:** Classical 3D Roughness Parameters: Arithmetic Mean Roughness (*S*_a_), Root Mean Square Roughness (*S*_q_), and Maximum Height Roughness (*S*_z_) of Neat PLGA and PLGA/β-TCP Composite Scaffolds

Experimental Groups	*S*_a_ (μm)	*S*_q_ (μm)	*S*_z_ (μm)
PLGA	20.4	27.1	189.3
PLGA/β-TCP	75.6	87.1	405.9

Meanwhile, the results of the topographic analysis at the nanometric scale, considering an area of 25 μm^2^, are graphically represented in Figure 8 and numerically in Table 2. Similar to the optical profilometry data, significant differences were observed between the surfaces of the samples. The nanoroughness of the PLGA/β-TCP composite scaffold was also higher for the 2D parameters of arithmetic mean roughness (*R*_a_), root-mean-square roughness (*R*_q_), and maximum roughness (*R*_max_), reaching increases of 620%, 105%, and 100%, respectively, when compared to the neat PLGA scaffold.

**Table 2:** Classical 2D Roughness Parameters: Arithmetic Mean Roughness (*R*_a_), Root Mean Square Roughness (*R*_q_), and Maximum Roughness (*R*_max_) of Neat PLGA and PLGA/β-TCP Composite Scaffolds

**Experimental Groups**	*R*_a_ (nm)	*R*_q_ (nm)	*R*_max_ (nm)
PLGA	3.2	4.3	63
PLGA/β-TCP	6.2	8.8	126

**Figure 8:**
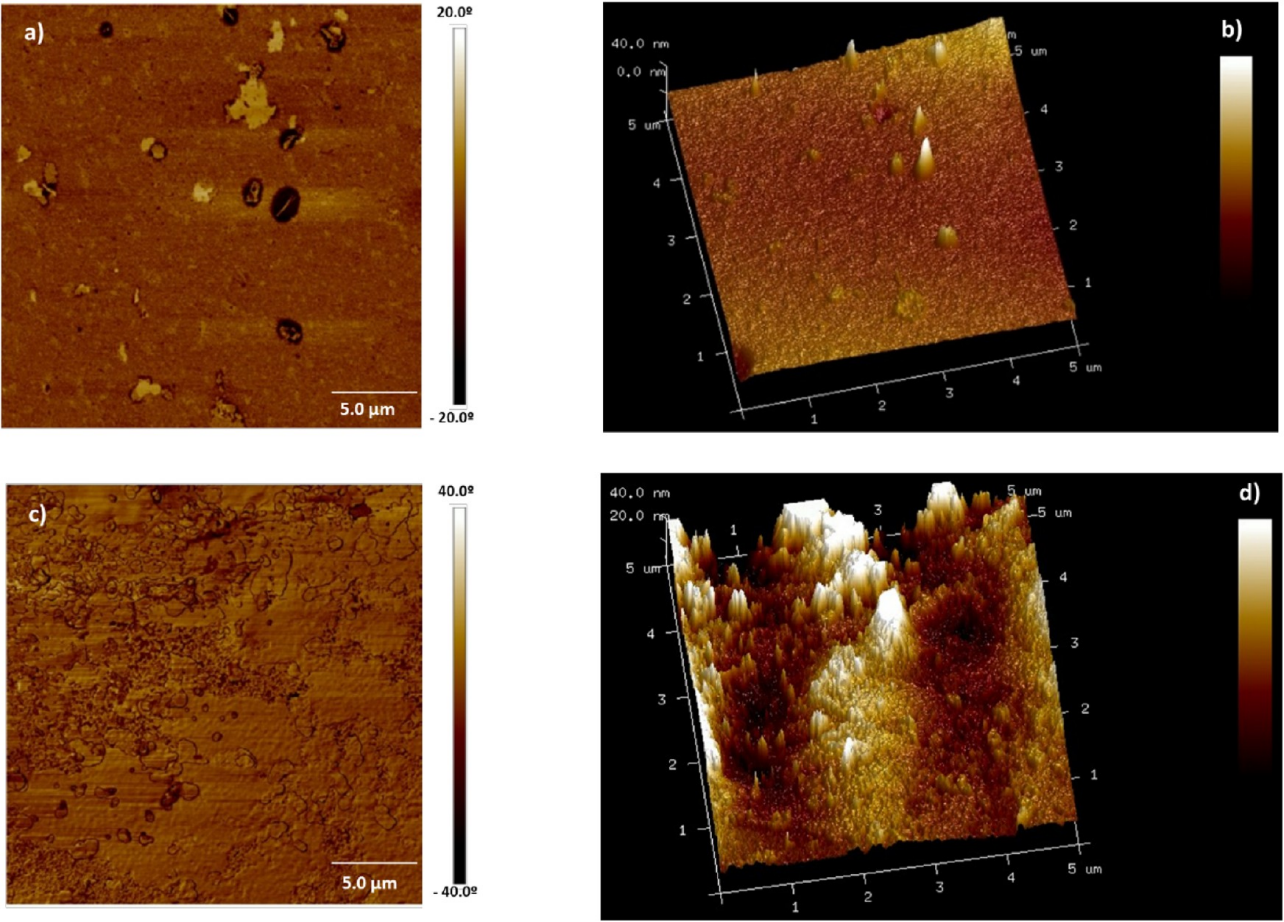
Topographic images obtained by AFM of the neat PLGA (a and b) and PLGA/β-TCP composite (c and d) scaffolds in two-dimensional presentation (a and c) and after three-dimensional topographic reconstruction (b and d) in an area of 5 μm × 5 μm. Images of the same samples in 3D dimension (b and d).

Considering the classic roughness parameters mentioned above (*S*_a_, *R*_a_, *S*_q_, *R*_q_, *S*_z_, and *R*_max_) at the micro- and nanometric scales, PLGA/β-TCP presented significantly higher values compared to pure PLGA. Zhao^43^ analyzed the behavior of MG-63 cells in their interaction with rough surface substrates and reported greater cytocompatibility provided by this type of surface characteristic, reinforcing the importance of this parameter for the development of scaffolds for use in the field of bone regenerative medicine (BRM).^44^

A more detailed evaluation of the 3D-printed scaffolds was performed using the parameters skewness (*S*_sk_), which describes the degree of symmetry or asymmetry of the surface height about the mean plane, and kurtosis (*S*_ku_), which indicates the nature of the height distribution; both are shown in Figure 9. It is important to note that negative *S*_sk_ values indicate plateau-type surfaces, while positive values correspond to surfaces with peak-dominated topography. *S*_ku_ values greater than 3 represent spiky surfaces, whereas values lower than 3 indicate bumpy features. In this context, the PLGA/β-TCP composite scaffold exhibited an opposite profile compared to PLGA, featuring a plateau-type surface with spaced, rounded peaks and valleys. Moreover, the surface of the PLGA/β-TCP composite can be described as approaching an *S*_sk_ Gaussian profile, meaning it presents a symmetric roughness distribution with material concentrated around the mean plane. These characteristics may be associated with the ceramic granules embedded in the neat PLGA matrix at the surface of the PLGA/β-TCP composite, which likely contribute to the formation of this plateau-like surface geometry. According to Saltzman and Kyriakides,^45^ the extent and shape of these cell-material contact regions directly impact cellular behavior, mediating events such as adhesion, spreading, and differentiation.

**Figure 9:**
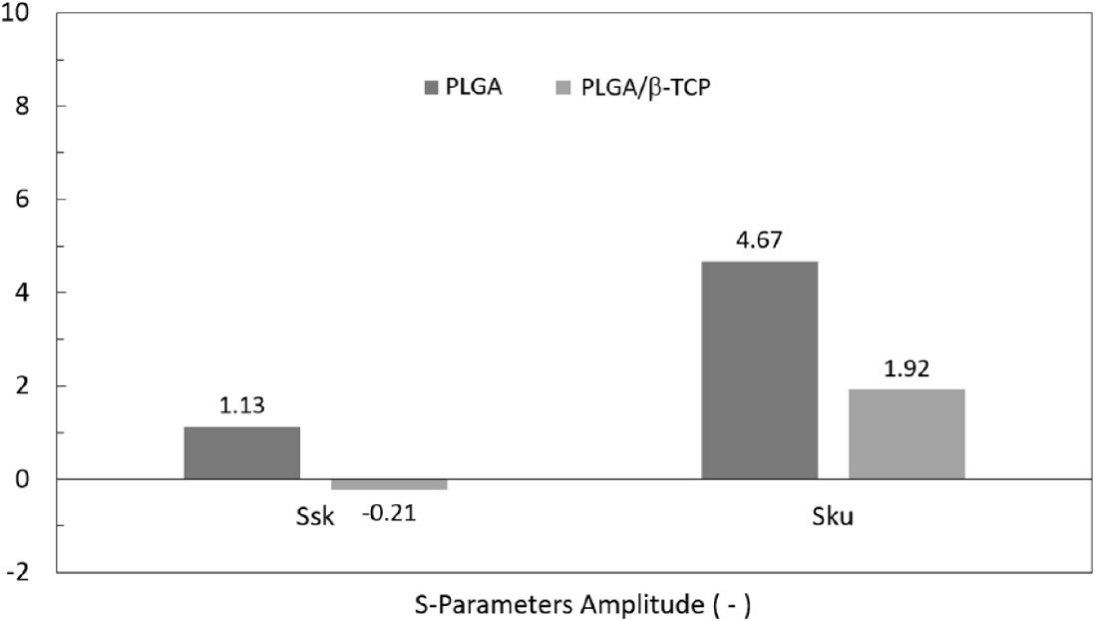
3D symmetry roughness (*S*_sk_) and distribution (*S*_ku_) of profile heights of neat PLGA and PLGA/β-TCP composite scaffolds.

In a secondary analysis, hybrid parameters (SI and *S*_dr_) and functional volumetric parameters (*S*_c_, *S*_v_, and *V*_n_) were used, with the respective values presented in Table 3. Initially, the ratio between the real surface area (three-dimensional profile) and the projected area of the samples was calculated, corresponding to the surface index (SI) parameter, which allows for assessing the degree of surface roughness. In percentage terms, the PLGA/β-TCP composite exhibited a 9.37% higher value than the neat PLGA scaffold. The developed interfacial area ratio (*S*_dr_), which represents the SI value normalized by the projected area of the real surface on the base plane of the profilometric image, was 4.9 times greater than that of the neat PLGA scaffold.

**Table 3:** Analysis Parameters Surface Index (SI), Developed Interfacial Area (*S*_dr_), Normal Volume (*V*_n_), Core Void Volume (*S*_c_), and Surface Void Volume (*S*_v_) of the Neat PLGA and PLGA/β-TCP Composite Scaffolds

Experimental Groups	SI (−)	*S*_dr_ (μm^–2^)	*V*_n_ (mm^3^/in^2^)	*S*_c_ (μm^3^/μm^2^)	*S*_v_ (μm^3^/μm^2^)
PLGA	1.09432	4680.58	57.07	38.84	1.86
PLGA/β-TCP	1.19688	22938.11	88.97	102.38	6.37

Addressing the normal volume parameter (*V*_n_), which indicates the volume between two surfaces parallel to each other and to the base plane—corresponding to the surface’s volumetric capacity—the PLGA/β-TCP composite exhibited a 55.9% higher capacity compared to the neat PLGA polymer. In order to further analyze and characterize this aspect, *V*_n_ was subdivided into three height intervals of roughness, parallel to the base plane of the profilometric image and considering the sample’s cross-sectional area (Figure 10).

**Figure 10:**
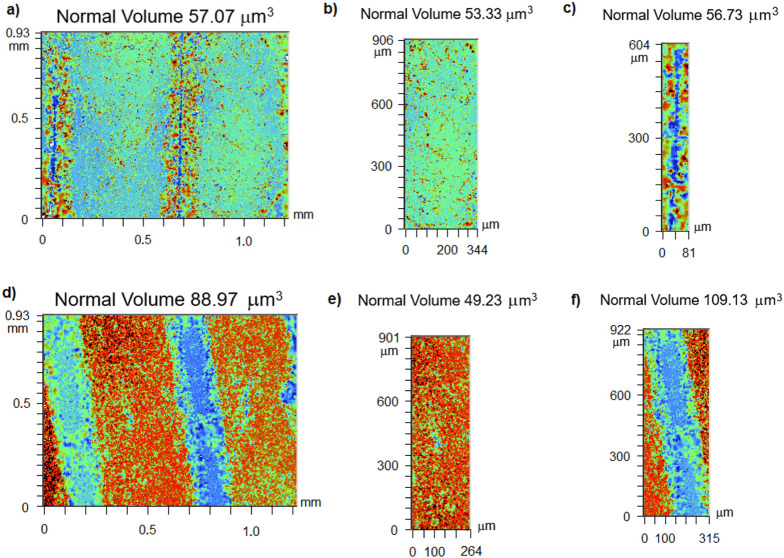
Normal volume of the entire surface of neat PLGA (a) and PLGA/β-TCP composite (d) scaffolds; normal volume of the plateaus of neat PLGA (b) and PLGA/β-TCP composite (e) scaffolds; normal volume of the channels of neat PLGA (c) and PLGA/β-TCP composite (f) scaffolds.

It allowed for the determination of the core void volume (*S*_c_), representing the volumetric capacity between 10% and 80% of the surface height, and the surface void volume (*S*_v_), corresponding to the volumetric capacity between 80% and 100% of the surface height. The PLGA/β-TCP composite scaffold presented values 164% and 242% higher for *S*_c_ and *S*_v_ parameters, respectively.

With the inclusion of the *S*_ds_ parameter, which reflects the number of summits (peaks) per unit area on the surface—that is, an increase in the amplitude and number of these peaks may also indicate a greater surface area—it was observed that the PLGA/β-TCP composite exhibited 1056.02 μm^–2^ of summits, while the neat PLGA presented 705 μm^–2^, corresponding to a percentage difference of 49.8% between the two experimental groups.

In summary, the parameters presented above (SI, *S*_dr_, *V*_n_, *S*_c_, *S*_v_, and *S*_ds_) provide three-dimensional insights and allow for comprehensive surface area analysis. It can be confirmed that the PLGA/β-TCP composite scaffold showed higher values compared to the neat polymer in each of these parameters, suggesting that it provides a greater contact surface. This characteristic promotes the adsorption of proteins present in bodily fluids, which are responsible for mediating the cell–material adhesion process.^46^

Finally, the *S*_tr_ parameter was used to assess whether or not there is directionality in the surface textures. In this context, values close to 0 indicate directional roughness, while values near 1 correspond to isotropic surfaces, meaning those that do not depend on direction. As shown numerically in Figure 11, the PLGA/β-TCP composite scaffold tends toward anisotropy, strongly influenced by the wider and deeper channels previously described in Figure 7. In contrast, the neat PLGA scaffold tends to present an isotropic surface. To complement the information provided by the *S*_tr_ parameter, *S*_al_ represents the horizontal distance related to the greatest decay of the autocorrelation function of the surface roughness. Accordingly, the PLGA/β-TCP composite exhibited a distance 6.4 times greater than that of the neat PLGA scaffold. It can be stated that every 88.75 μm of asperity spacing corresponds to statistical changes in the surface texture direction, which confirms its anisotropic nature. According to Ribeiro,^47^ materials with anisotropic surface characteristics influence cell behavior by modulating their morphology and stimulating osteogenic differentiation.

**Figure 11:**
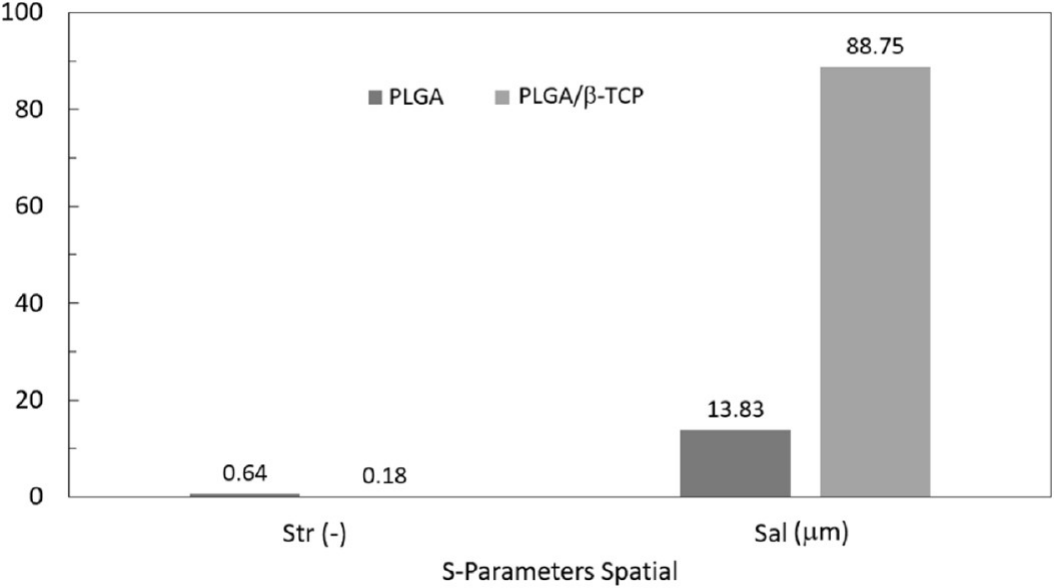
Texture aspect ratio (*S*_tr_) and length relative to the largest decay of the autocorrelation function (*S*_al_) of neat PLGA and PLGA/β-TCP composite scaffolds.

The micro- and nanoscale surface modifications observed in the PLGA/β-TCP composite scaffold, as evidenced by the results presented, demonstrate the impact of ceramic incorporation into the neat PLGA polymer scaffold and its potential influence on cytocompatibility. It is worth emphasizing that cytocompatibility is a critical factor in evaluating the osteointegration potential of an engineered bone substitute developed through BRM strategies.^44^ In summary, the results presented above are based on parameters that constituted a broad and detailed topographic analysis of the scaffolds. It is also noteworthy that some of the surface parameters employed had not yet been explored in the scientific literature for polymeric and ceramic materials for this application. Nevertheless, they allowed for an expanded understanding of the topographic characteristics that are determinant for cytocompatibility and osteointegration in the context of regenerative bone medicine applications.

### 3.4 Characterization of the Mechanical Properties of Neat PLGA and PLGA/β-TCP Composite Scaffolds

The analysis of the surface mechanical properties of neat PLGA and PLGA/β-TCP composite scaffolds, based on the parameters of adhesion, DMT modulus, and height, is presented in Table 4 and Figure 12 and reveals significant differences between the scaffolds.

**Table 4:** Mechanical Property Values Obtained by PeackForce Quantitative Nanomechanical Mapping (QNM) Techniques^a^

Experimental groups	Adhesion (nN)	DMT modulus (GPa)	Height (nm)
PLGA	20.0	3.0	20.0
PLGA/β-TCP	10.0	4.0	420.0

a The evaluation of adhesion, DMT modulus, and height for the neat PLGA and PLGA/β-TCP composite.

**Figure 12:**
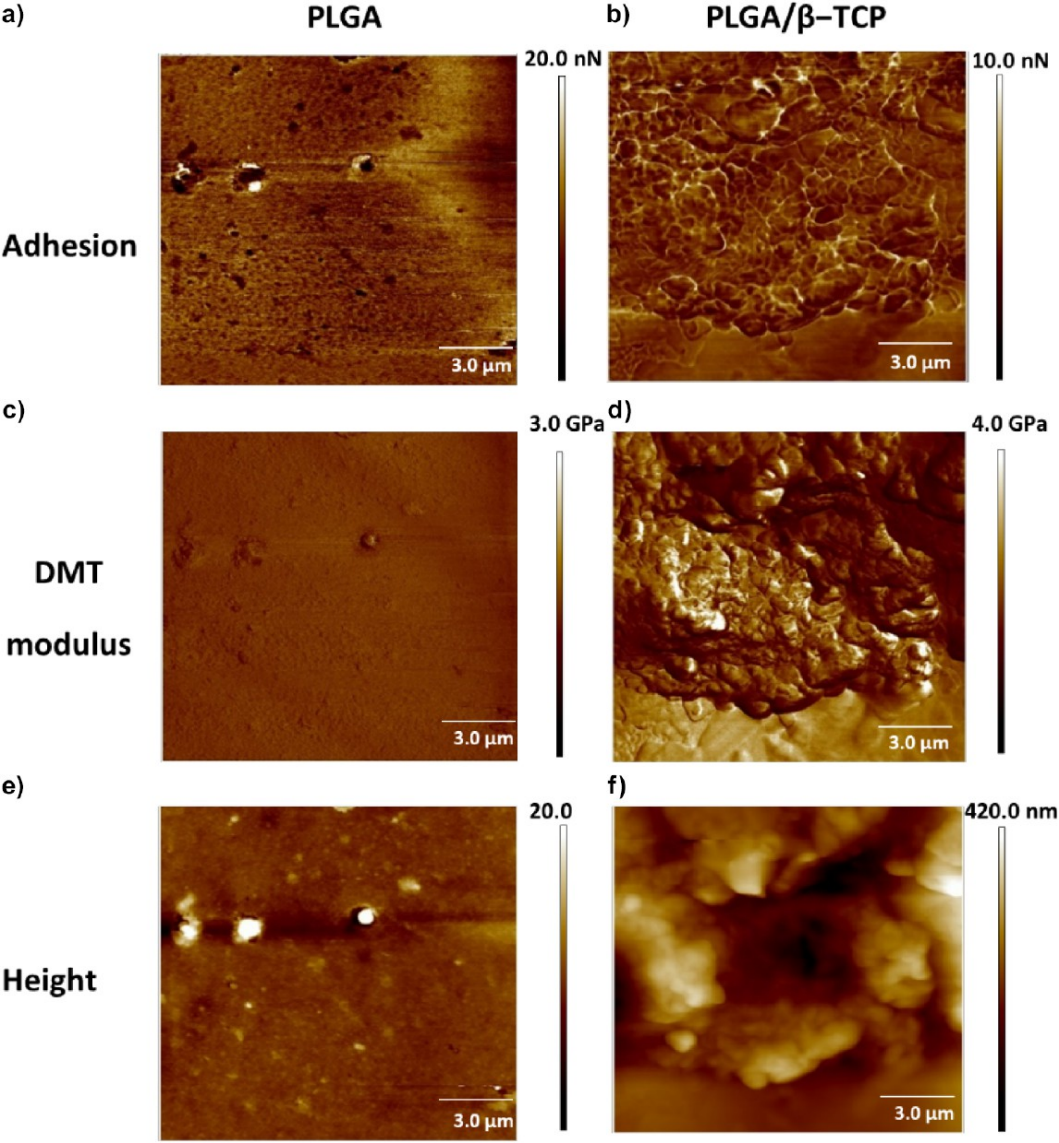
PeackForce QNM images of adhesion (a,b), DMT modulus (c,d), and height (e,f) of neat PLGA (a,c,e) and PLGA/β-TCP composite scaffolds (b,d,f).

The Young’s modulus, measured using the DMT model, showed lower variability in the neat PLGA scaffold, with average values ranging from 0.5 to 2.5 GPa, indicating greater homogeneity in surface stiffness distribution. In contrast, the incorporation of β-TCP particles resulted in a wider range of modulus values. It increased the overall stiffness of the composite scaffold compared to the neat PLGA scaffold, with values ranging from 0.5 to 4.5 GPa.

It becomes evident that the presence of β-TCP granules contributed to the increased stiffness of the composite scaffold, with higher elastic modulus values than the neat PLGA scaffold. The difference in values between 2.5 and 4.5 GPa is likely attributable to the ceramic component at the scaffold surface. Notably, the minimum required modulus for scaffolds intended for trabecular bone applications is approximately 5 GPa, which is close to the value detected for the PLGA/β-TCP composite.^48^ As highlighted by Belgin et al.,^49^ the mechanical properties of the substrate strongly influence both the adhesion and osteogenic differentiation processes, with the latter playing a key role in conferring osteoinductive properties to the scaffold.

### 3.5 Cytocompatibility Test

Quantitative and qualitative analyses related to the cytocompatibility assay are shown in Figures 13 and 14. Within the first 24 h of the experiment—corresponding to the cell adhesion phase—the PLGA group exhibited reduced cell viability compared to the other experimental groups, with an average viability of 79%. In contrast, the PLGA/β-TCP group showed an average viability of 96%, a value very similar to the control group, considered the ideal environment for cell interaction (100%). These differences were confirmed through statistical analysis. On the second day of the experiment—associated with the cell proliferation phase—an increase in viability was observed across all groups. However, unlike the 24 h of analysis, the control and PLGA/β-TCP groups no longer displayed analogous values, showing increases of 77% and 62%, respectively. When comparing the experimental groups, a proportional increase in viability was detected, with the PLGA group reaching 63%.

**Figure 13:**
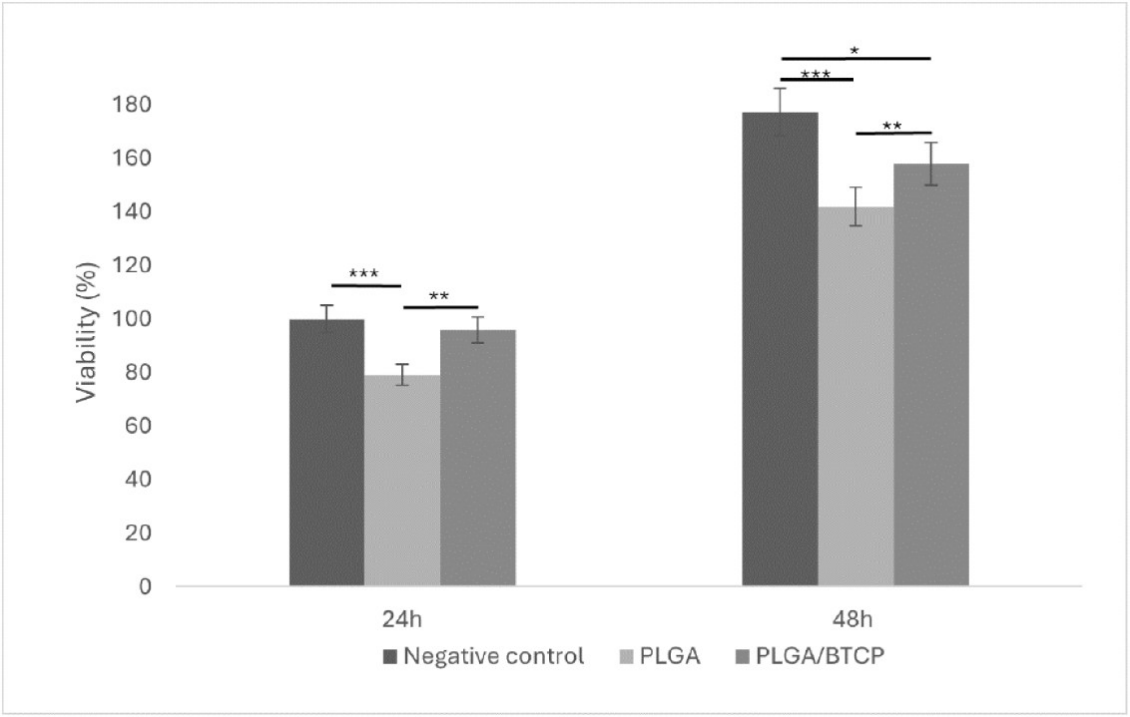
Viability assay of the human osteosarcoma cell line (MG-63) with the resazurin assay. Analyses were performed in the control group, neat PLGA scaffold, and PLGA/β-TCP composite scaffold at 24 and 48 h. Statistical analysis was performed using one-way ANOVA, considering a significance level of 5% (*p* < 0.05). * *p* ≤ 0.05, ** *p* < 0.003, *** *p* < 0.000.

**Figure 14:**
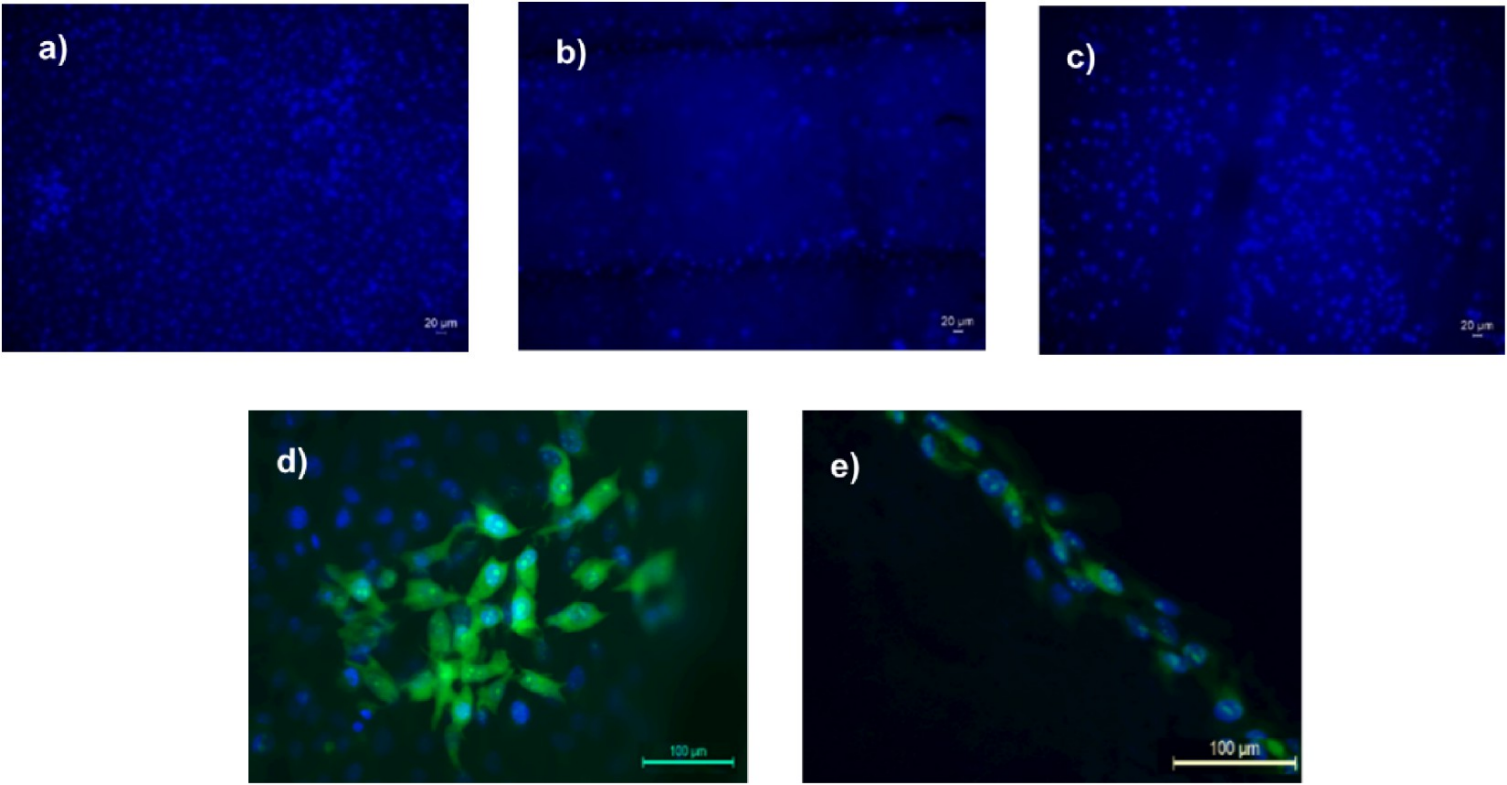
Fluorescence microscopy photomicrograph determines the qualitative analysis of cell viability, which allows visualization of the cell nuclei in the negative control (a), neat PLGA (b), and PLGA/β-TCP composite scaffolds (c). The neat PLGA scaffold (b) was able to preserve cell viability for 24 and 48 h; however, the PLGA/β-TCP composite scaffold (c) proved to be more attractive to the cells, as it was closer to the ideal cell survival conditions than the negative control group (a). Images (d: 100 μm) and (e: 100 μm) allow visualization of the cell cytoplasm (green) and nuclei (blue) in the neat PLGA (d) and PLGA/β-TCP composite (e) scaffolds.

Considering the biocompatible characteristics of neat PLGA, it was observed that the scaffold fabricated from this polymeric material exhibited an increase in cell viability over both days of analysis. This result can be supported by the study of Fahimipour,^24^ which demonstrated the polymer’s high affinity for cells. Nonetheless, the cytocompatibility values of the PLGA sample were 17% lower when compared to the control and PLGA/β-TCP groups, which, according to statistical analysis, presented analogous values. This outcome may be associated with the lower bioactivity of PLGA, which is known to improve when ceramics such as β-TCP are incorporated.^10^ The properties of β-TCP are reinforced by the findings of Khojasteh,^19^ who confirmed that cells are capable of adhering to and proliferating on scaffolds composed solely of this ceramic material, likely due to its osteogenic potential. Furthermore, Parisi et al.^25^ demonstrated that the interaction between cells and materials occurs through the interface of adsorbed proteins and the material surface, a process strongly influenced by the physicochemical and surface characteristics of the scaffold.

Considering all the previously presented characterization results of the PLGA/β-TCP scaffold, it is possible to conclude that the presence of β-TCP modified the conditioning of the material, resulting in several distinct surface features. These modifications significantly impacted cellular behavior. It is worth noting that the presence of the ceramic phase may positively influence osteogenic differentiation and stimulate bone matrix deposition.^50^ However, these aspects were not the focus of the present study.

## 4 Conclusion

This study enabled physicochemical, morphological, topographical, and surface mechanical characterization, as well as the cytocompatibility analysis of neat PLGA and PLGA/β-TCP biodegradable polyester/ceramic composite scaffolds. The results demonstrated that the presence of β-TCP significantly increased surface roughness at both the micro- and nanoscale. The prominent values of parameters such as *S*_a_, *S*_q_, *S*_z_, *V*_n_, and SI, and the distribution statistics *S*_sk_ and *S*_ku_, along with *R*_a_, *R*_q_, and *R*_max_ for the composite scaffold, revealed a more complex surface with a predominance of smooth, rounded peaks and valleys. Moreover, this substrate exhibited greater surface stiffness, approaching values considered suitable for bone substitutes. These findings can be correlated with the positive impact on the cytocompatibility profile of the β-TCP-containing sample, particularly within the first 24 h of analysis, considering the increased occurrence of cell adhesion phenomena to the biomaterial.

Therefore, it can be inferred that the 3D-printed PLGA/β-TCP scaffolds exhibit topographical features that positively influence cytocompatibility, an essential aspect for osseointegration. These findings contribute to the understanding of the biological performance of biodegradable polyester/ceramic composites and reinforce their application in tissue engineering and bone regenerative medicine. Nevertheless, further studies are required to explore the impact of nonconventional surface parameters (*S*_sk_, *S*_ku_, *S*_dr_, *S*_al_, SI, *S*_tr_, *V*_n_, *S*_c_, *S*_v_, and *S*_ds_) on cytocompatibility. Future investigations may also include more in-depth analyses of cellular behavior, such as protein adsorption and osteogenic differentiation, in order to assess the osteoinductive potential of the material.

## ∇

Rafaela B. Silvestre: São Carlos Institute of Physics (IFSC), Av. Trabalhador São Carlense, 400, São Carlos—SP, Brazil; Biosmart Nanotechnology—Av. Jorge Fernandes de São Mattos, 311, Araraquara—SP, Brazil

## ◆

Marcos A. S. Gutiérrez: Biofabrication Laboratory-Divisão DTPS, Renato Archer Information Technology Center (CTI), CP 13069-901, Campinas—SP, Brazil.

## Abbreviations

β-TCP: Beta-Tricalcium Phosphate
3D: Three-Dimensional
CAD: Computer-Aided Design
CBCs: Critical-Size Bone Defects
DMEM: Dulbecco’s Modified Eagle Medium
XRD: X-ray Diffraction (formerly DRX in Portuguese)
FTIR: Fourier Transform Infrared Spectroscopy
GBR: Guided Bone Regeneration
SEM: Scanning Electron Microscopy (formerly MEV in Portuguese)
AFM: Atomic Force Microscopy
MG-63: Human Osteosarcoma Cell Line
RBM: Regenerative Bone Medicine
PBS: Phosphate-Buffered Saline
PLA: Poly­(lactic acid)
PLGA: Poly­(lactic-*co*-glycolic acid)
FBS: Fetal Bovine Serum
TGA: Thermogravimetric Analysis
